# Monopolar electrocautery versus sharp dissection in the neck dissection: a retrospective study

**DOI:** 10.1038/s41598-023-31328-x

**Published:** 2023-03-16

**Authors:** Katharina Theresa Obermeier, Paris Liokatis, Wenko Smolka

**Affiliations:** Department of Oral and Maxillofacial Surgery and Facial Plastic Surgery, University Hospital, LMU Munich, University of Munich, LMU, Lindwurmstr. 2a, 80337 Munich, Germany

**Keywords:** Cancer, Surgical oncology, Head and neck cancer, Oral cancer

## Abstract

The cold scalpel/scissors (CS) and the monopolar electrocautery (ME) are still the most commonly used instruments for neck dissection in head and neck oncology. However, a direct comparison of these techniques does not exist. This study aims to compare these techniques concerning blood loss, the decline of hemoglobin levels, and surgery duration. Data on 200 patients who received tumor resection, neck dissection and either a radial forearm free flap (RFFF)or a primary closure (PC) were examined retrospectively. The patients were divided according to the performed defect closure (RFFF or PC) and the main instrument usedfor the beck dissection (Group 1: RFFF and ME, Group 2: RFFF and CS, Group 3: PC and ME Group 4: PC and CS). The intraoperative blood loss, decline of hemoglobin values and surgery duration were analyzed and compared between the corresponding groups. The patients where the ME was used lost on average 409.93 ml (group 1 vs. 2) and 242.4 ml (group 3 vs. 4) less blood. The median decrease in the hemoglobin levels was by 1.01 g/dL (group 1 vs. 2) and 0.85 g/dL (group 3 vs. 4) lower for the ME. The median surgery duration was by 102 min (group 1 vs. 2) and 83 min (group 3 vs. 4) shorterfor the ME. All differences were statistically significant. Traditional scalpel and scissors used for neck dissection lead to significantly higher blood loss and longer operation time than the monopolar electrocautery.

## Introduction

Oral squamous cell carcinoma (OSCC) counts with 95% to the most common cancer of the oral cavity^1^. Surgical resection of the primary tumor is still the first-line treatment for OSCC^2^. Because of the 20–40% risk for tumor spreading in the locoregional lymph nodes, guidelines in many cases recommend not only resection of the primary tumor but also removal of the cervical lymph nodes^3,4^.

Although neck dissection is a trivial procedure in head and neck surgery,the literature discusses implementing several instruments and methods to improve the intraoperative process and postoperative results. Among them, surgical instruments such as monopolar electrocautery (ME) and ultrasonic or harmonic scalpel (US) are already used and studiedin many surgical fields and procedures^5–7^. Implementing these techniques aims to decrease the intraoperative bleeding and trauma in the tissues, leading to reduced operating times and perhaps a shorter hospitalization of patients and overall better postoperative outcome. However, these energy instruments maybe are associated with an increased risk of healing disturbances due to the additional thermal trauma caused in some tissues^7^.

In head and neck surgery, the above-mentioned modern instruments have also been in use. Although several studies compare the harmonic scalpel to traditional scalpel and scissors and other energy instruments in the dissection of the neck^8–13^, there is to our knowledge no study comparingthe two basic techniques most widely used for soft tissue dissection in the neck: the monopolar electrocautery and the cold scalpel or scissors.

Hence, this retrospective case–control study aims to evaluate and compare monopolar electrocautery toconventional scalpel and scissors used forneck dissection in head and neck oncology. The two techniques are compared concerning blood loss, decline of the hemoglobin (Hb) levels and surgery duration, two important reasons for implementing these modern instruments.

## Material and methods

This study was approved by the institutional ethic committee of the Ludwig Maximilian University of Munich, Germany (Munich, Germany, ref. number: 20-1096). All methods were performed in accordance with the guidelines and regulations of this journal. Informed consent was obtained from all subjects and/or their legal guardians.

The present retrospective case-controlstudy includes patients who underwent surgical tumor resection due to an OSCC and simultaneous neck dissection between 2013 and 2019. Exclusion criteria were a history of a coagulation disturbance, dissection or radiotherapy in the neck area. Furthermore, patients who suffered from extensive soft tissue or bone defects after the tumor resection, which were reconstructed with other than a radial forearm free flap (RFFF), were also excluded to achieve homogenous groups. Moreover, patients who received additional surgeries other than tumor resection and neck dissection during the main procedure were also excluded.

Finally, 200 patients were included in the study. All neck dissections were performed by six surgeons experienced in head and neck surgery. All patients received adrenaline injections prior to the first incision. To achieve comparable and homogenous groups, the patients were divided initially into two groups according to the performed defect closure: patients who underwent tumor resection, neck dissection and reconstruction with anRFFF and those who received tumor resection, neck dissection and primary closure (PC) of the defect. Each initial group was further divided into two groups according to the main instrument usedto dissect the cutaneous and subcutaneous tissue of the neck (monopolar electrocautery or cold scalpel/scissors) as documented in the surgeons' report. As a result, four final groups were defined and compared:

- Group 1 (RFFF and ME) versus Group 2 (RFFF and CS)
- Group 3 (PC and ME) versus Group 4 (PC and CS)

In order to investigate for possible inhomogeneity between the groups, the tumor diameter and localization as documented in the pathology report, the extent of neck dissection, the preoperative coagulation parameters (international normalized ratio-INR and activated partial thromboplastin time-aPTT) and hemoglobin levels were considered.

### Outcome measures

The primary outcome parameters examined were duration of surgery, decrease of the hemoglobin levels and estimated blood loss. The blood loss was calculated by measuring the amount in the suction and weighing the gauzes. The postoperative hemoglobin value was evaluated two days after surgery to avoid falsification due to infusions during surgery. Information about intra- and postoperative blood transfusions was also collected.

### Statistical analysis

Statistical analysis was conducted using SPSS® 24 version 4.0 (SPSS Inc., Chicago, IL, USA). The results calculated and compared for the four groups were the median duration of the surgery, median blood loss and the median differenceof the pre-and postoperative Hb levels. The Shapiro–Wilk-Test was used for determining the distribution pattern of the data, and they were found not normally distributed.

To search for statistical significance, the Mann–Whitney-U-Test was applied. Statistical significance was defined as *p* < 0.05. Additionally, we calculated the effect size r.

## Results

Of the 200 patients included, 92 patients were females and 108 males. The mean age of the patients at the first diagnosis was 63.75 years, ranging from 30 to 88 years.

The site of the tumor resection was reconstructed at 48 patients with the use of an RFFF. For the remaining 152 patients, primary closure (PC) of the defect was performed.

Among the 48 patients in the RFFF group, the monopolar electrocautery (ME) was used to dissect the neck's soft tissues in 30 cases, while in 18 cases, the cold scalpel or scissors (CS) were the main instruments.

In the PC group,the neck dissection was performed at 95 patients with electrocautery and 57 patients with the conventional scalpel/scissors.

The patient's demographics, tumor dimensions/localization, extent of neck dissection, and preoperative coagulation values (INR and aPTT) were documented (Table 1). In all four groups, most of the tumors were located in the mouth floor and tongue (groups 1 and 2:66.7 and 55.6% respectively and groups 3 and 4: 67.4 and 64.9% respectively). The most frequently performed neck dissection was in levels I–III at both sides (groups 1 and 2: 66.7 and 73.3% respectively and groups 3 and 4: 68.4 and 77.2% respectively). Furthermore, no great differences were found regarding the tumor’s diameter (groups 1 and 2: 23.5 mm (5–45 mm) and 25.5 mm (11–59 mm), respectively, and groups 3 and 4: 19 mm (6–59 mm) and 18 mm (5–65 mm), respectively). The median INR values were for groups 1 and 2: 1.03 (0.9–1.2) and 1.07 (0.9–1.3), respectively, and for groups 3 and 4: 1.04 (0.9–1.4) and 1.07 (0.9–1.5), respectively. The median apTT values were also similar: for groups 1 and 2: 35.8 s (21–36 s) and 30.0 s (28–40 s), respectively and groups 3 and 4: 30.4 s (26–36 s) and 30.8 s (21–40 s), respectively. In conclusion, no great preoperative differences were found between the corresponding groups regarding the dimensions and localization of the primary tumor, the extent of neck dissection and the coagulation parameters.

**Table 1:** Characteristics of the four groups regarding the primary tumor, the extent of neck dissection and coagulation parameters.

Transfusions medizinisch			RFFF group		PC group	
			Group 1 (monopolar, n = 30)	Group 2 (scalpel, n = 18)	Group 3 (monopolar, n = 95)	Group 4 (scalpel, n = 57)
Extent of neck dissection	I–III bothsides		73.3% (22)	66.7% (12)	68.4% (65)	77.2% (44)
	I–V bothsides		13.3% (4)	27.8% (5)	9.5% (9)	8.8% (5)
	I–III ipsilateral + I contralateral		13.3% (4)	8.3% (1)	19% (18)	14% (8)
	Other		0	0	3.2% (3)	0
Primary tumor	Lokalisation	Floor-Tongue	66.7% (20)	55.6%(10)	67.4% (64)	64.9% (37)
		Buccal plane	23.3% (7)	16.7%(3)	10.5% (10)	10.5% (6)
		Lower lip	0	0	4.2% (4)	7% (4)
		Upper jaw	6.7% (2)	16.7%(3)	11.6% (11)	12.3% (7)
		Other	3.3% (1)	11.1%(2)	6.3% (6)	5.3% (3)
	Median diameter (mm)		23.5	25.5	19	18
Median Coagulation values	INR		1.03 (0.9–1.2)	1.07 (0.9–1.3)	1.04 (0.9–1.4)	1.07 (0.9–1.5)
	apTT		35.8 (21–36)	30.0 (28–40)	30.4 (26–36)	30.8 (21–40)

### Hemoglobin levels

#### Group 1 (RFFF and ME) versus group 2 (RFFF and CS)

The preoperative median hemoglobin values in groups 1and 2 were 13.4 g/dL (range: 9.3–16 g/dL) and 13.4 g/dL (range 11.1–15.8 g/dL), respectively. The postoperative values were 10.7 g/dL (range: 8–14.6 g/dL) for group 1 and 9.69 g/dL (range: 7.6–15.8 g/dL) for group 2. As a result, the median drop of the Hb levels was 2.7 g/dL for group 1 and 3.71 g/dL for group 2. By comparing both groups, the statistical analysis showed that the decrease in the Hb levels differ significantly between groups 1 and 2, with the patients operated with the cold scalpel/scissors having an increased drop in the Hb value (*p* = 0.048). The effect size (r < 0.5) showed a low effect for this test.

#### Group 3 (PC and ME) versus group 4 (PC and CS)

**Figure 1:**
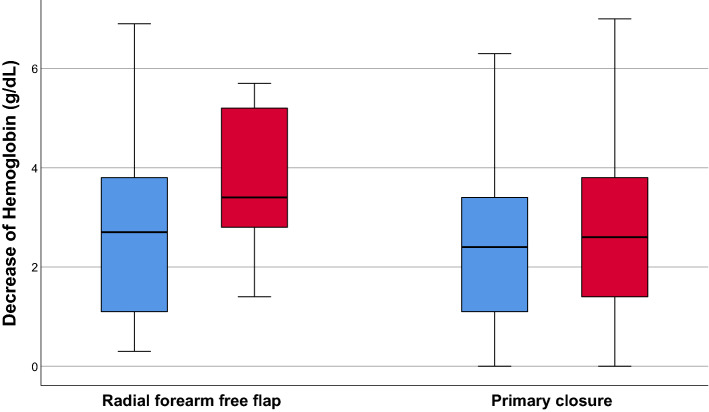
Decrease of Hemoglobin.

The hemoglobin values for groups 3 and 4 were preoperatively 13.72 g/dL (range: 9.3–16.7 g/dL) and 13.1 g/dL (range: 10.2–15.6 g/dL), respectively, and postoperatively 11.27 g/dL (range: 7.7–15.7 g/dL) and 9.8 g/dL (range: 7.6–13.8 g/dL), respectively. The median decrease in the hemoglobin levels after the surgery was 2.45 g/dL for group 3 and 3.3 g/dL for group 4, with a statistically significant difference (*p* = 0.014) and a strong effect (r > 0.5). The results of hemoglobin decrease are shown in Fig. 1.

### Blood loss

#### Group 1 (RFFF and ME) versus group 2 (RFFF and CS)

The estimated median blood loss in group 1 was 330.67 ml (range: 150–800 ml), while in group 2 was 740.6 ml (range: 170–1800 ml). This shows a median difference of 409.93 ml concerning the blood loss between both groups, which was statistically significant (*p* = 0.01) with a strong effect (r > 0.5) of the test.

#### Groups 3 (PC and ME) and 4 (PC and CS)

**Figure 2:**
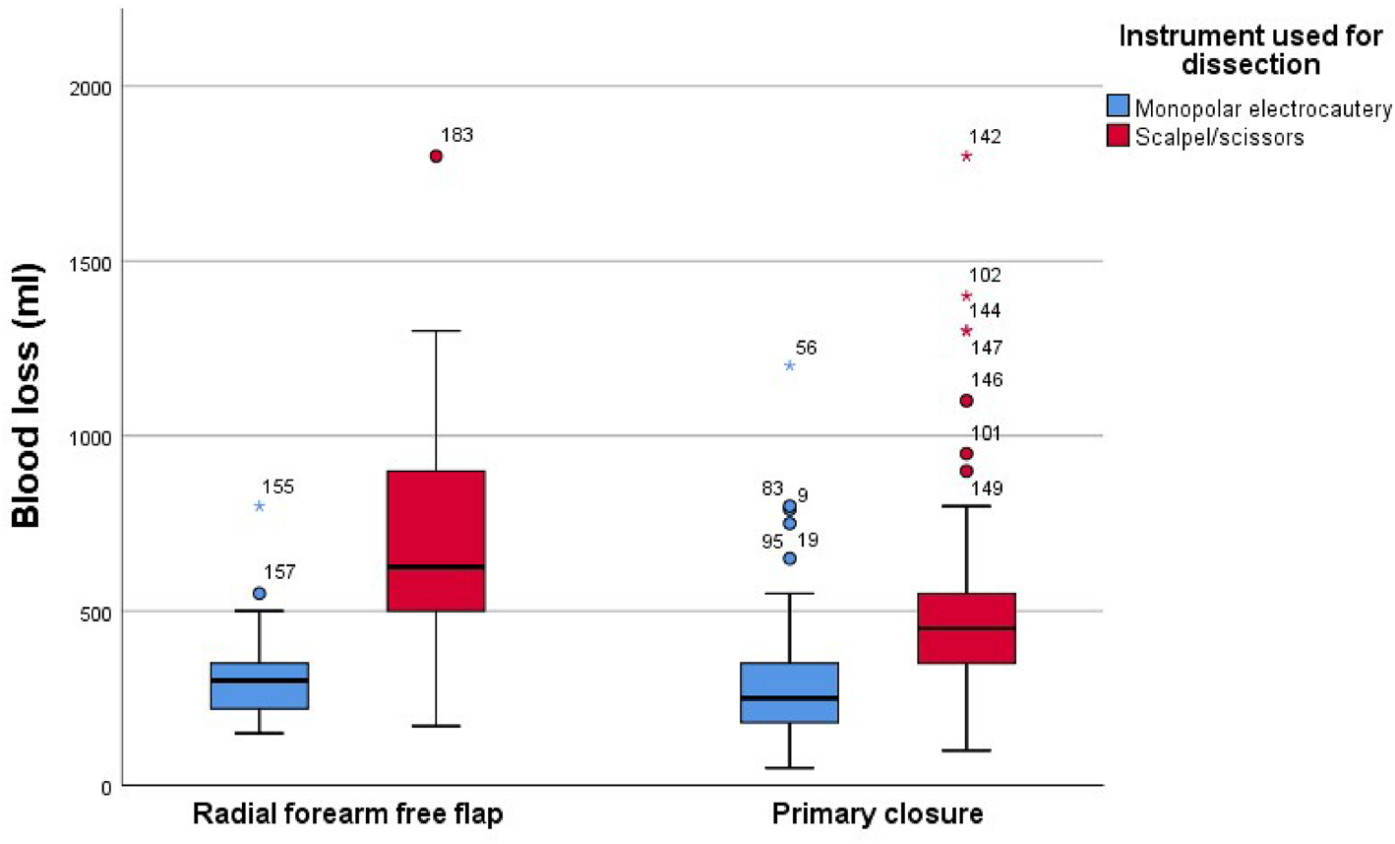
Estimated blood loss.

The estimated median blood loss was 283.6 ml (range: 50–1200 ml) for group 3 and 526 ml (range: 150–1800 ml) for group 4. The median difference of the blood between groups 3 and 4 was 242.4 ml and was statistically significant (*p* = 0.001) with a strong effect (r > 0.5). The results of blood loss of both groups are shown in Fig. 2.

### Duration of surgery

#### Groups 1 (RFFF and ME) and 2 (RFFF and CS)

The median duration of the surgery was for group 1: 418 min (range: 251–750 min.) and for group 2: 520 min (300–710 min.) with a statistically significant difference (*p* = 0.032) and a low effect (r < 0.05) of the test.

#### Groups 3 (PC and ME) and 4 (PC and CS)

**Figure 3:**
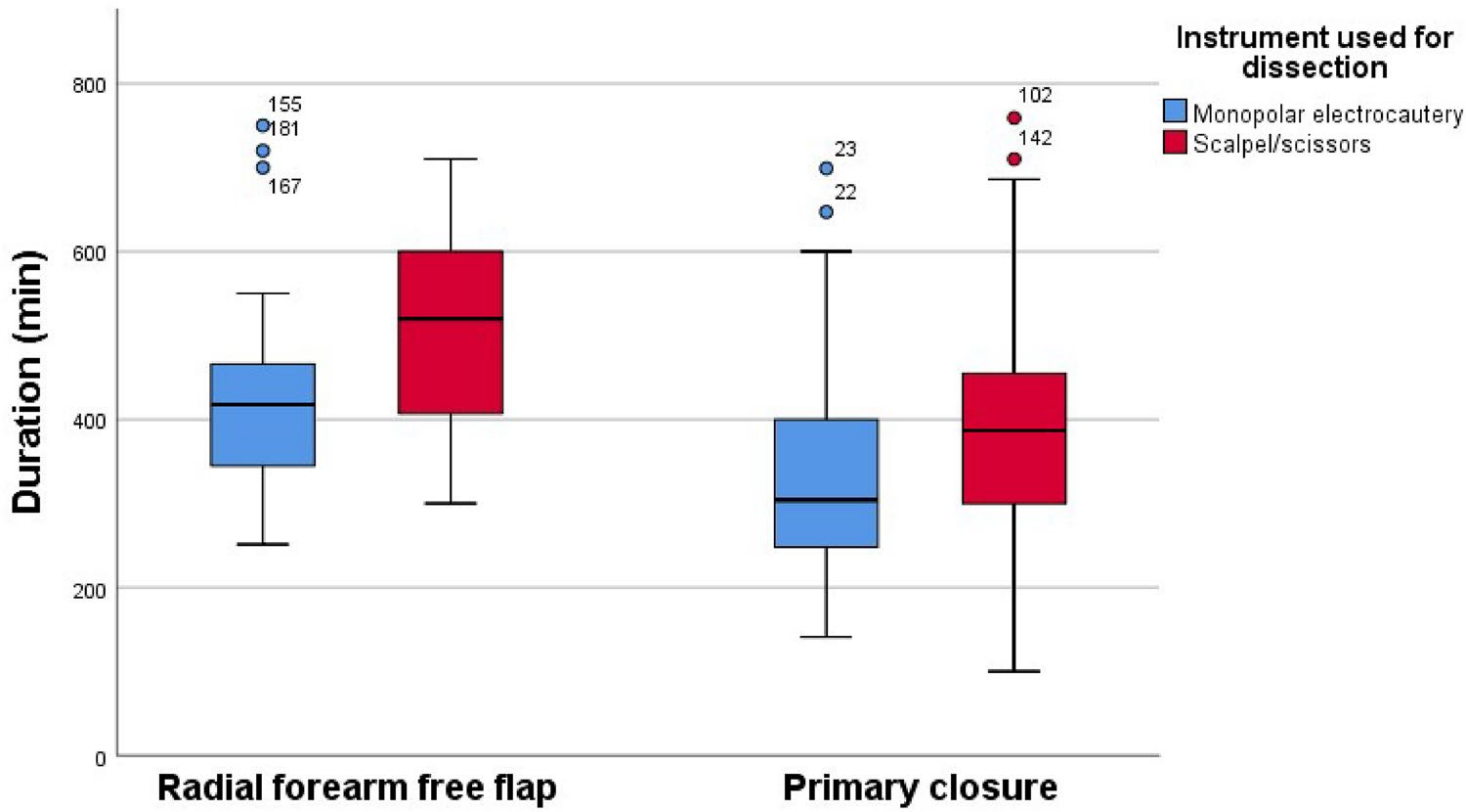
Duration of surgery.

The median duration of surgery was 304 min (141–647 min.) for group 3 and 387 min (130–759 min.) for group 4, with a statistically significant difference (*p* = 0.02) and a low effect (r < 0.05). The results of duration of surgery are shown in Fig. 3.

### Blood transfusions

Blood transfusions were performed on six patients (2.7%) (Table 2). Two patients belong to group 1 and four patients to group 2. One erythrocyte concentrate was administered in four patients, two concentrates in one patient, and three concentrates in the last patient.

**Table 2:** Overview of patients who received erythrocyte concentrates.

Patient	Group	Hb preop. (g/dl)	Hb postop. (g/dl)	Hb-decrease (g/dl)	Number of EC received
1	1	15.6	8.7	6.9	1
2	1	11.3	9.6	1.7	1
3	2	11.1	9.0	2.1	1
4	2	15.1	9.4	5.7	1
5	2	12.9	9.8	3.1	2
6	2	12.8	8.0	4.8	3

## Discussion

Modern surgical instruments such as the harmonic scalpel and the monopolar electrocautery are evaluated in the dissection of various tissues and organs in many fields^6,7^. Τhese energy instruments are becoming more popular in head and neck surgery and are compared to each other with various and sometimes contradicting results^11–14^. However, the cold scalpel and scissors and the monopolar electrocautery remain the mainstream techniques for neck dissection, but no direct comparison between them exists.

In the current study, the patients received either a sharp neck dissection with the cold scalpel/scissors or dissection with the monopolar electrocautery. The patients in the groups where the ME was used compared to the corresponding groups with the sharp dissection lost on average 409.93 ml (group 1 vs. group 2) and 242.4 ml (group 3 vs. group 4) less blood. Moreover, the median decrease in the hemoglobin levels was by1.01 g/dL for group 1 versus group 2 and 0.85 g/dL for group 3 versus group 4 higher for the patients who received a sharp dissection. The measured decline in the hemoglobin levels agrees with the estimated blood loss considering the suction and gauzes since it is known that loss of one unit of blood results approximately in a decrease of 1 g/dL on hemoglobin levels. Regarding the surgery duration, in groups 1 and 3 operated with the ME, the surgery lasted on average 102 and 83 min shorter than in groups 2 and 4, respectively, where the scalpel/scissors were used. All differences mentioned above were statistically significant and in favor of using the monopolar electrocautery instead of the traditional instruments. Concerning the intraoperative blood loss and the reduced surgical times, ou results are similar to the findings from studies regarding tonsillectomy and thyroid surgery^15,16^ and indicate a clear advantage of monopolar electrocautery compared to the traditional instruments.

The shorter operation time using the ME is probably due to the fact that ME immediately coagulates smaller bleedings, too. A shorter operation time may lead to decreased risk for postoperative delirium^17^ and may affect the hospitalization and the patient's postoperative quality of life^18^. Hasegawa et al.^19^ found that older age, extensive surgical procedures, more prolonged operation, excessive bleeding, and blood transfusion may lead to more extended postoperative management in the intensive care unit, a longer hospital stay and postoperative delirium. This means that patients who had surgery performed with ME may have a lower risk for postoperative delirium and a better quality of life.

However, it is known that the ME is elevating the temperature in the surrounding tissues. Using electrocautery, the temperature of 200 °C at the tip leads to carbonization of the neighboringtissueand causes an additional thermal trauma^20^, which may lead to increased postoperative pain and healing disturbances in some tissues^21,22^. The harmonic scalpel seems to have further advantages to the monopolar electrocautery^21^, although the cases’ heterogeneity does not offer safe conclusions for the neck dissection^10^.

All six patients (2.7%) who received transfusions with erythrocyte concentrates had received reconstruction with an RFFF. By four patients was used the CS, and by two the ME. Although the general threshold for performing a transfusion against anemia is a Hb value of 7–8 g/dL, the final decision should be individualized and based on a complete evaluation of the patient's clinical condition and an assessment of the perfusion and oxygenation of vital organs through laboratory and clinical parameters^23^.

Overall this study still has some limitations. The study design is retrospective. In order to collect data based on this study in the future, a prospective design should be selected. The present study did not consider thermal tissue damage that may result from electrosurgery. Also, postoperative pain was not evaluated. In a randomized clinical trial, Schneider et al. compared the intensity of postoperative pain in harmonic scalpel and ME^21^. The harmonic scalpel turned out to come with less postoperative pain. Our study collected no data about postoperative pain or quality of life for comparing the two surgical techniques.

In conclusion, prospective randomized studies should be performed based on this and other retrospective studies to compare both surgical techniques.

## Data Availability

The datasets generated and/or analysed during the current study are not publicly available due but are available from the corresponding author on reasonable request, because during data evaluation process all data has been anonymised.
